# Supplementary material for: Whole blood immunophenotyping uncovers immature neutrophil-to-VD2 T-cell ratio as an early marker for severe COVID-19
Source: Nat Commun. 2020 Oct 16;11:5243. doi: 10.1038/s41467-020-19080-6 (PMC7568554; doi:10.1038/s41467-020-19080-6)
Supplement: Supplementary file 1 — Supplementary Information [file 41467_2020_19080_MOESM1_ESM.pdf]

Whole blood immunophenotyping uncovers immature neutrophil-to-VD2 T-cell ratio as an early marker for severe COVID-19

Carissimo G, Xu W, Kwok I *et al.*, 2020

Supplementary Figure 1

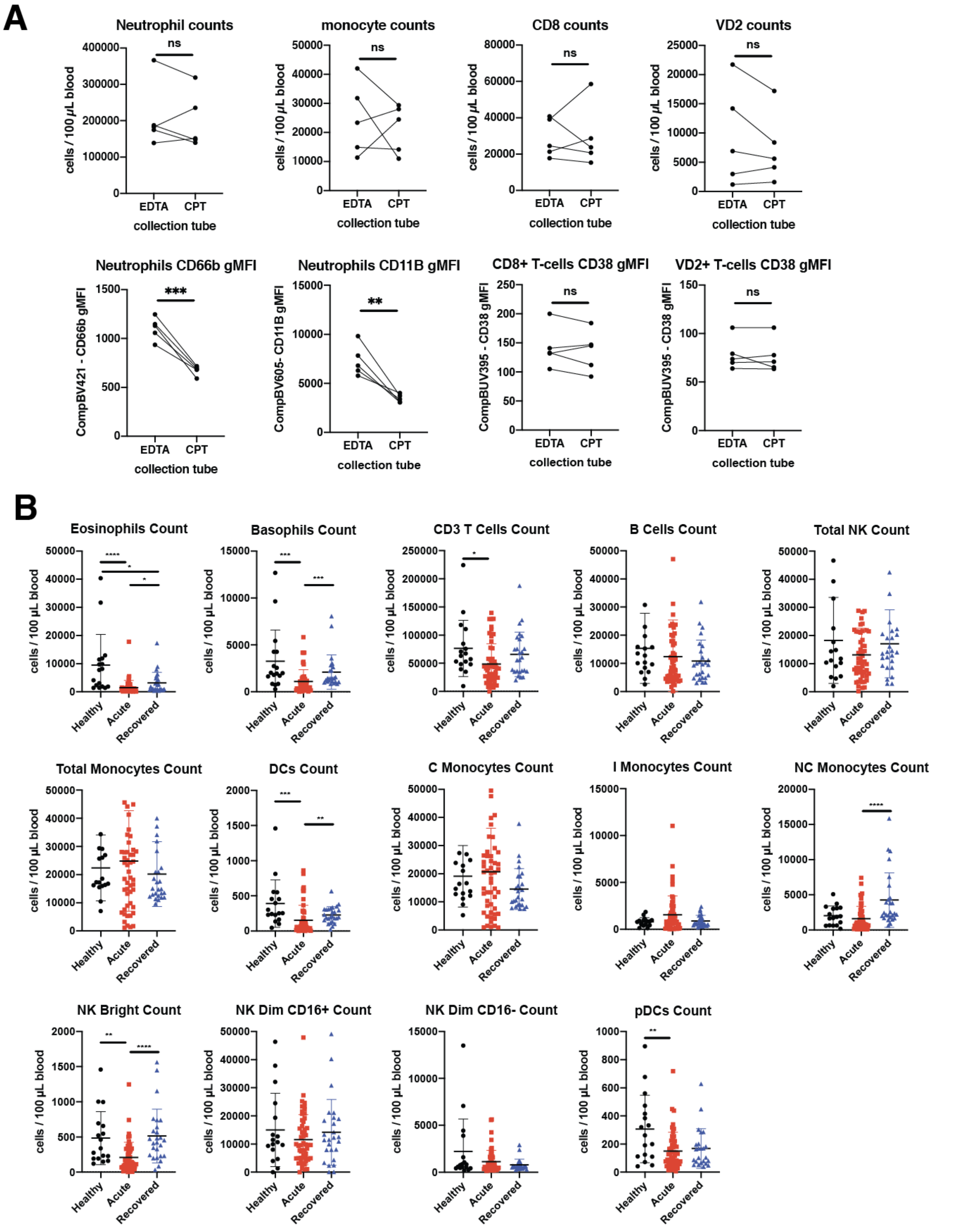

Supplementary Figure 1: (a) blood collection in CPT tubes affects phenotypic markers but not cell counts. Paired analysis was performed either with Wilcoxon non-parametric test two tailed for cell counts or ratio of paired t-test two tailed for gMFI n=5 individual donors (b) Individual plots for heatmap of cell counts presented in Figure 1b (healthy n=17, acute n=54, recovered n=26 unique individuals, from flow panels A and C). Scatter dot plots are presented with mean ± SD. Absolute counts were analysed by Kruskal-Wallis using Dunn correction for multiple comparison, *p<0.05, **p<0.01, ***p<0.001. Data available in source data file, exact p-values are given in Supplementary Table 4.

Supplementary Figure 2

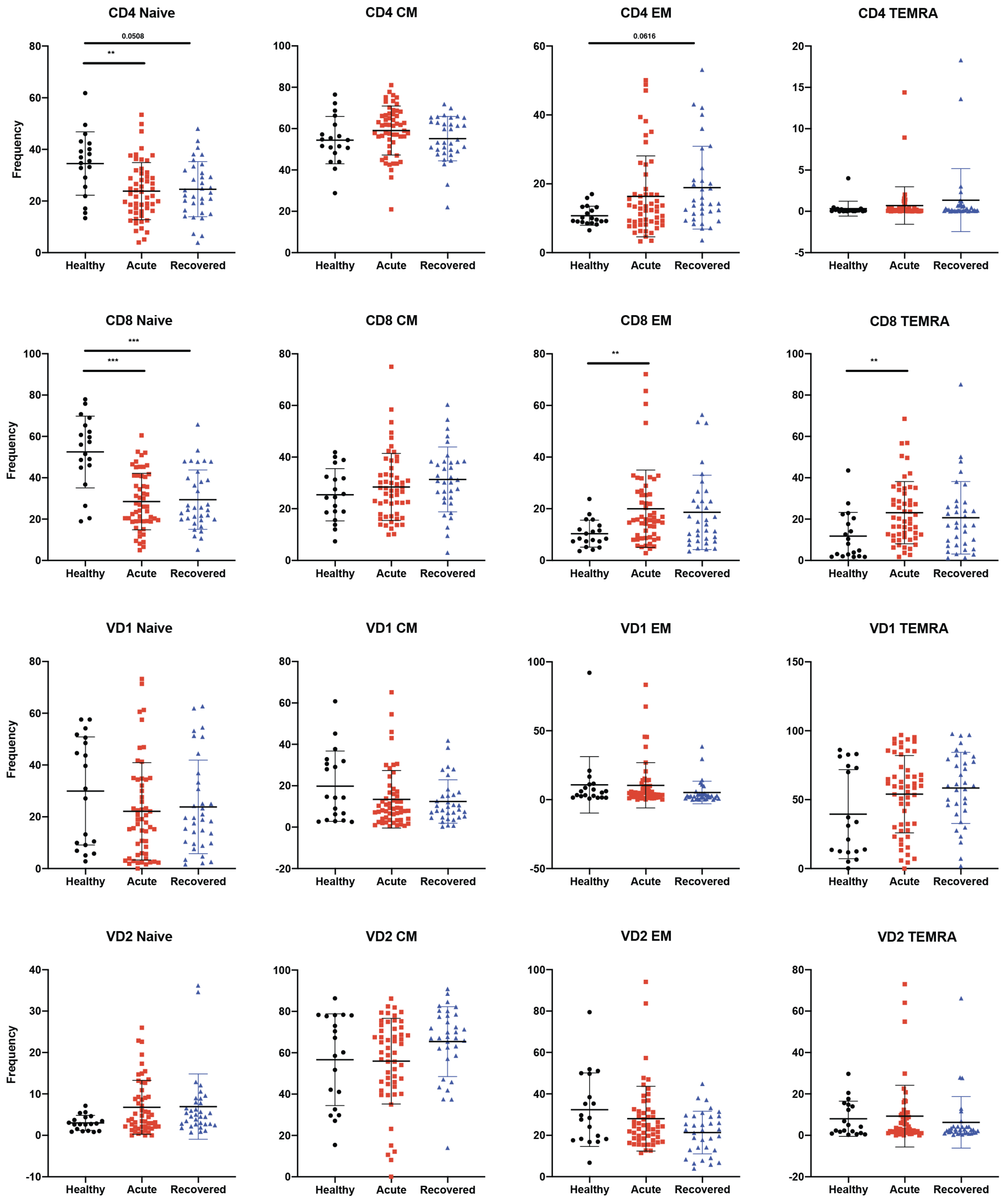

Supplementary Figure 2: Individual plots for heatmap of Figure 2d. Individual frequencies of CD45RA vs CD27 differentiation stage of CD8, CD4, VD1 and VD2 T-cells. Scatter dot plots are presented with mean ± SD. 1b (healthy n=19, acute n=54, recovered n=28 unique individuals, from panel B) Frequencies were analysed by Kruskal-Wallis using Dunn correction for multiple comparison. *p<0.05, **p<0.01, ***p<0.001. Data available in source data file, exact p-values are given in Supplementary Table 4.

Carissimo_Xu_Kwok el al. 2020

Supplementary Figure 3

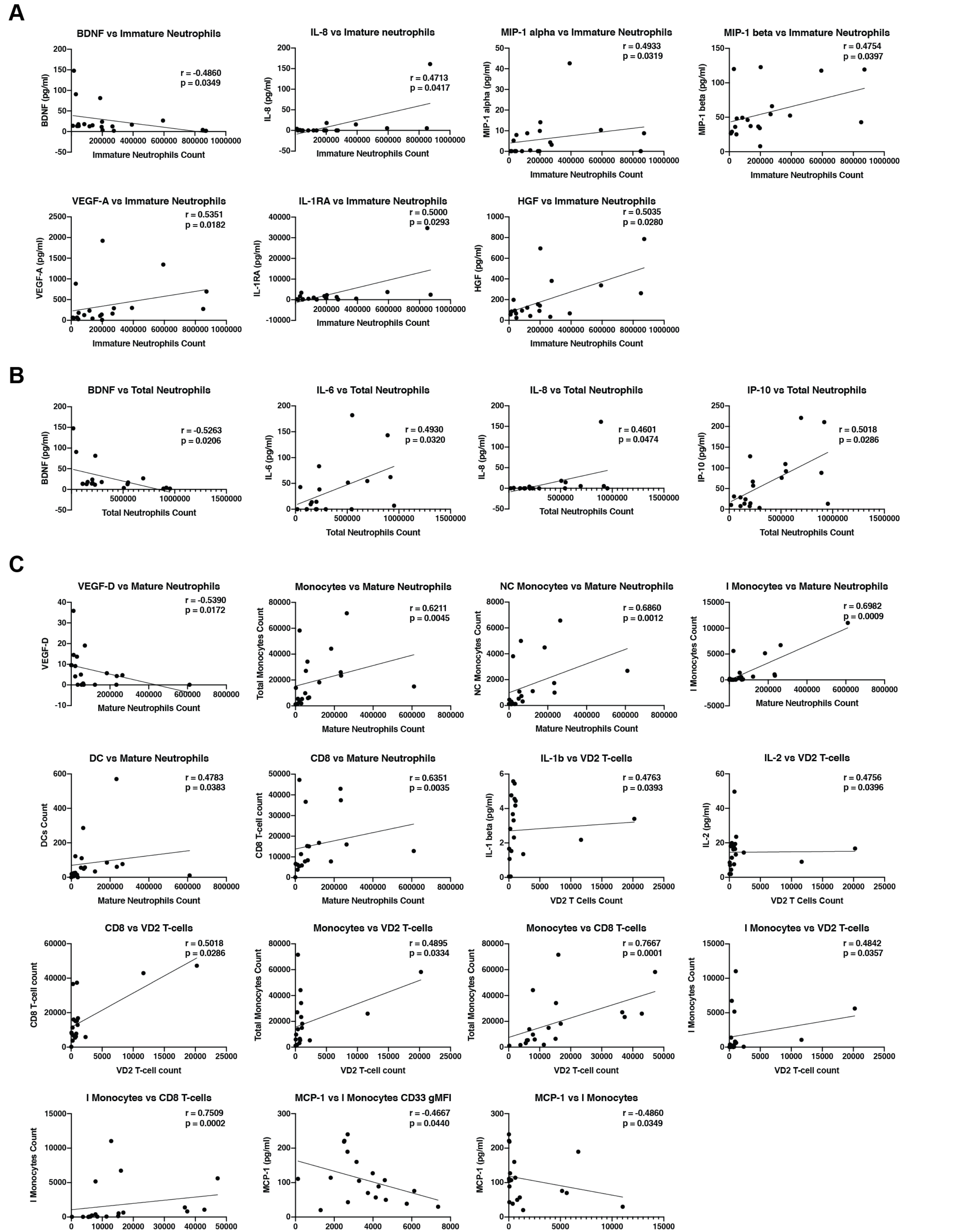

Supplementary Figure 3: Individual correlation plots for Figure 4. Non-parametric Spearman two-tailed test were performed for the parameters indicated in the plots for n=19 patients that had paired Luminex plasma readings (a) Individual plots of immature neutrophil counts with cytokines not presented in main Figure 4b. (b) Individual plots of immature neutrophil counts with cytokines. (c) correlations between other immune cells and cytokines. rho and p values of the correlations are indicated on the individual plots . Data available in source data file, exact p-values are given on each panel.

Supplementary Figure 4

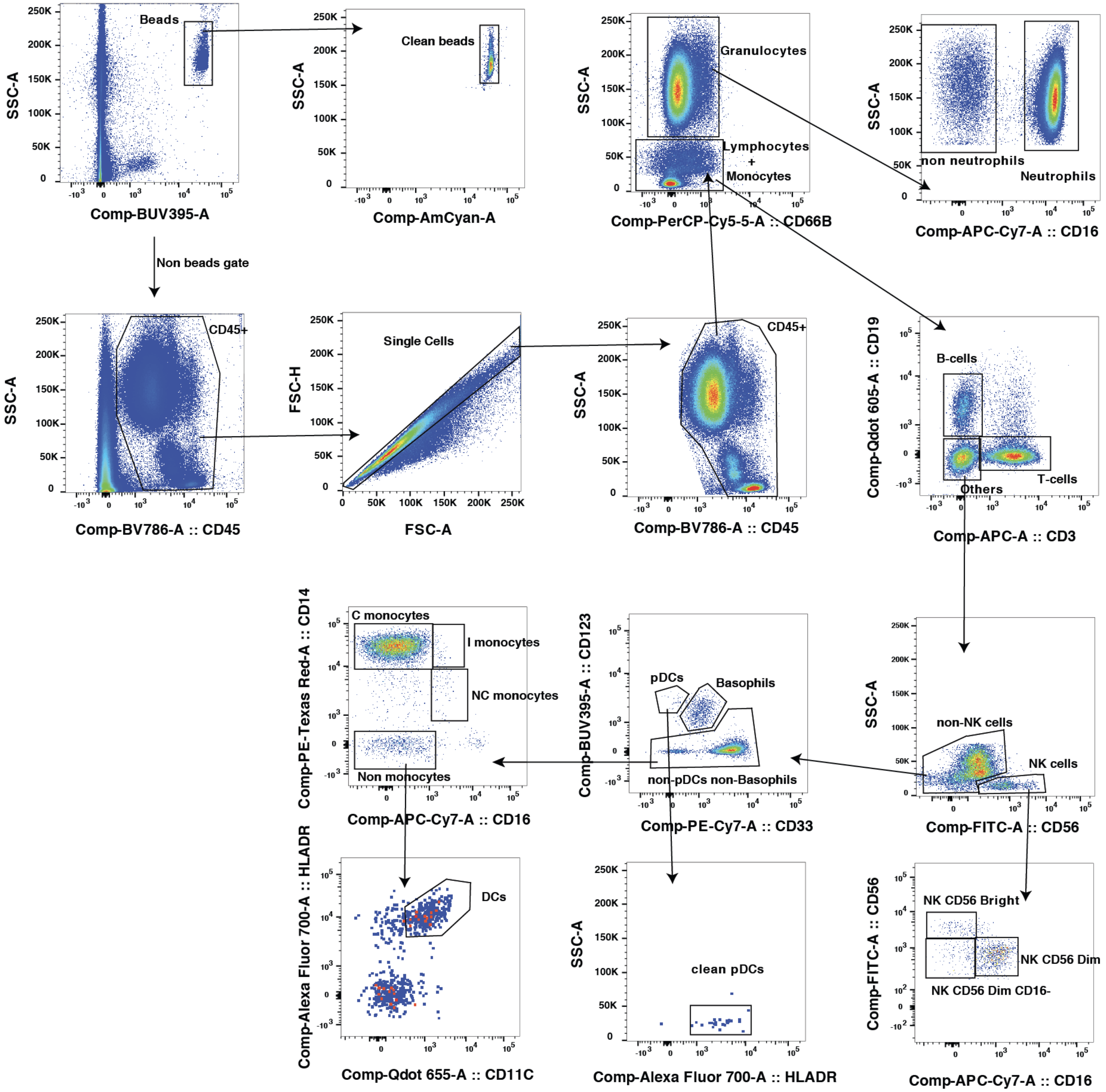

Supplementary Figure 4: Gating strategy for flow cytometry panel A. 100µL of blood was stained with the antibodies of panel A described in the supplementary table 3. Gating was performed after compensation adjustment in flowjo as presented in this figure. SSC-A vs CD45 allows identification of CD45+ population. From that, FSC-A vs FSC-H single cells are obtained, a second gating for ssc-a vs cd45 allow to clean the cd45+ population. Next high SSC-A cd66b+ population is the granulocytes from which can be gated the neutrophils as cd16+. From the low SSC-A population, CD19 vs CD3 allows to separate the B-cells (CD19+) and the T-cells (CD3+) and the double negative (DN) population. The DN population can then be used to gate the NK cells as SSC-A low, CD56+ population, those can the be separated into the CD56bright CD16- ; CD56int Cd16- and CD56int CD16+ populations. The non-NK cells can then be use to gate the CD33-CD123+ pDCs (which can then be cleaned into true pDCs as SSC-A low HLA-DR+) and the Basophils (CD33int, CD123+). The non-pDCs non-Basophils population can then be used to gate the Classical(CD14+CD16-)/intermediate(CD14+CD16+) and non-classical monocytes(CD14int CD16+) using CD14 vs CD16. The DC population (CD11c+HLA-DR+) can then be separated from the CD14-CD16- cells using CD11c vs HLA-DR. From all those population the activation markers can be investigated for % positive and gMFI. Data from this panel was used in Fig1b, 1c, 1d, 3c, 3d, Supp Fig 1a, 1b, 3c.

Supplementary Figure 5

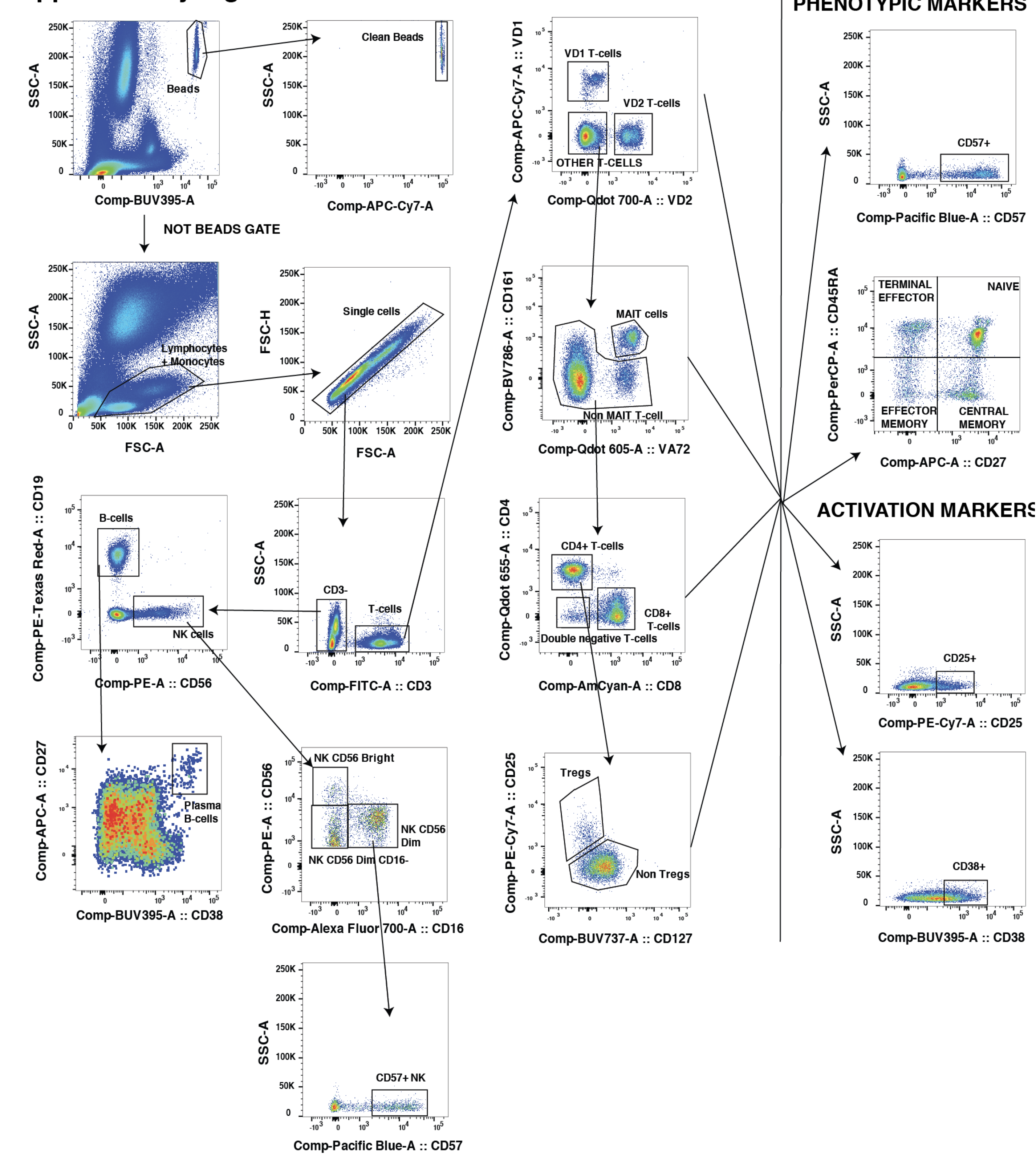

Supplementary Figure 5: Gating strategy for flow cytometry panel B. 100µL of blood was stained with the antibodies of panel B described in the supplementary table 3. Gating was performed after compensation adjustment in flowjo as presented in this figure. SSC-A low vs FSC-A allows to gate the lymphocyte and monocyte populations. From that, FSC-A vs FSC-H single cells are obtained. SSC-A low CD3+ T-cell population can then be gated. gamma delta T-cells can then be separated as VD1+ or VD2+. VD1-VD2- T-cells can be used to gate the MAIT cells (CD161+ VA7.2+) and the non-MAIT cells, which are then separated into CD4+ or CD8+ or DN T-cells. The CD4 T-cells can then be separate into Tregs (CD25+, CD127-) and effector CD4 T-cells (rest of the CD4+). From the CD3- population, we can gate the B-cells (CD19+) and the NK cells (CD56+). Plasmablast can be separated from the B-cell population using the CD38high Cd27high gate, while the NK population can ge divided in the 3 populations (as in panel A). Naive, memory and effector T-cell compartments can be investigated using the standard CD45RA vs CD27 quadrant methods. From all those population the activation and phenotypic markers can be investigated for % positive and gMFI. Data from this panel was used in Fig 2b, 2c, 2d, 3b, 5a, 5b, Supp Fig 1a, 2, 3c.

Supplementary Figure 6

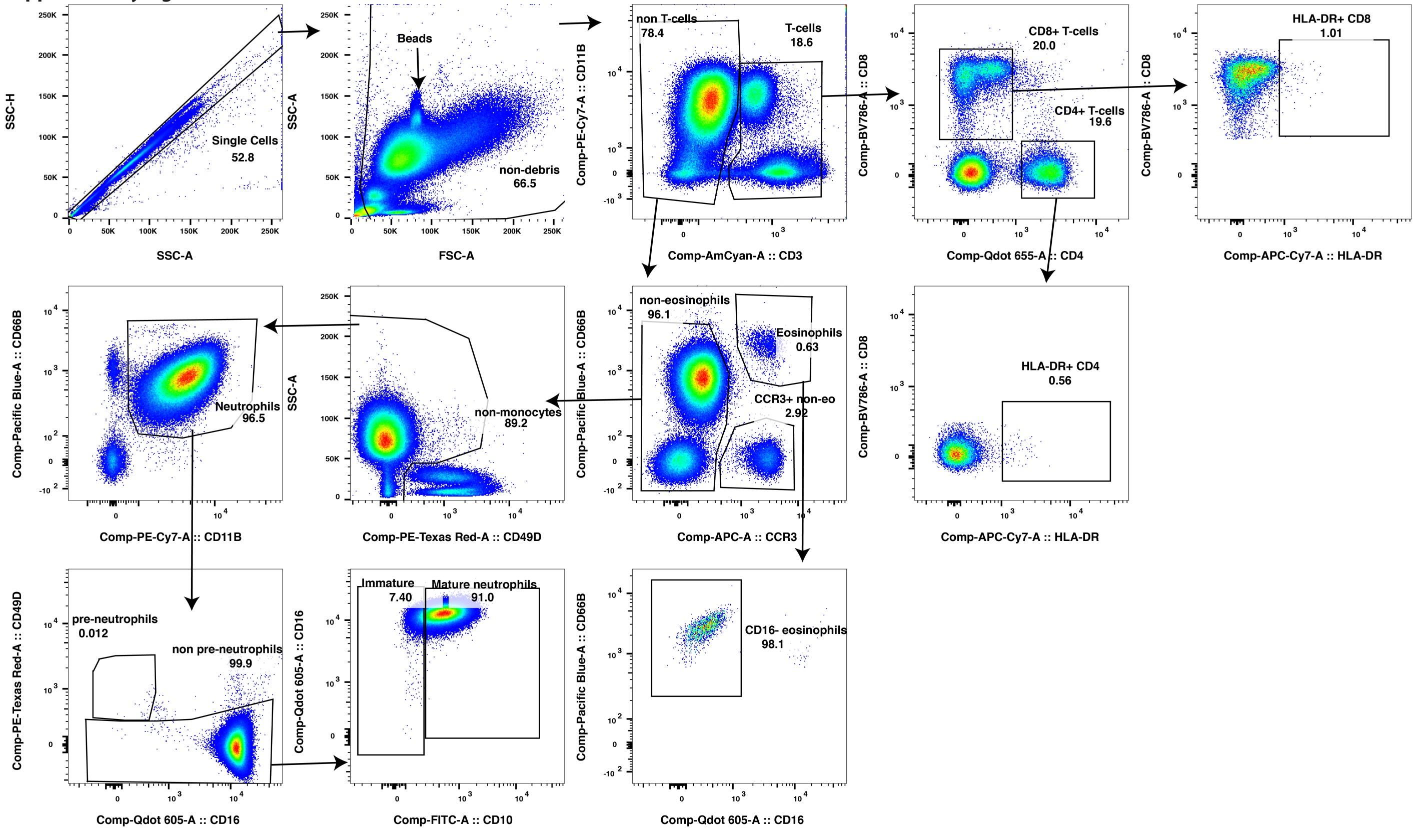

Supplementary Figure 6: Gating strategy for flow cytometry panel C. 100µL of blood was stained with the antibodies of panel C described in the supplementary table 3. Gating was performed after compensation adjustment in flowjo as presented in this figure. Single cells are first gated using the SSC-A vs SSC-H gate, and then cells are gated using the FSC-A and SSC-A gate. From that, the T-cells containing population (CD3+) and a non-T-cell containing population (CD3-) can be separated. In the CD3+ population, CD4+ and CD8+ T-cell population can be gated and their HLA-DR expression assessed. From the CD3- population, the CD66B vs CCR3 allows to gate the CCR3+CD66B- population, the CCR3+ CD66B+ eosinophil population (which is then cleaned in true eosinophils as the CD16- population), and the CCR3- population (non-eosinophil). These CCR3- cells and then gated as neutrophils in the CD66B+ CD11b+ gate. Next, pre-neutrophils can be gated as CD49dintCD16- and non pre-neutrophils can be gated as CD16+ CD49d-. These can then be separated using CD16 vs CD10 as mature neutrophils (CD10+) and immature neutrophils (CD10-). In this gate CD16 expression can be variable depending on the sample type (infected vs healthy). Data from this panel was used in Fig1b, 1e, 1f, 1g, 1h, 3d, 4a, 4b, 5a, 5b, Supp Fig 1a, 1b, 3a, 3b, 3c.

Supplementary Figure 7

A

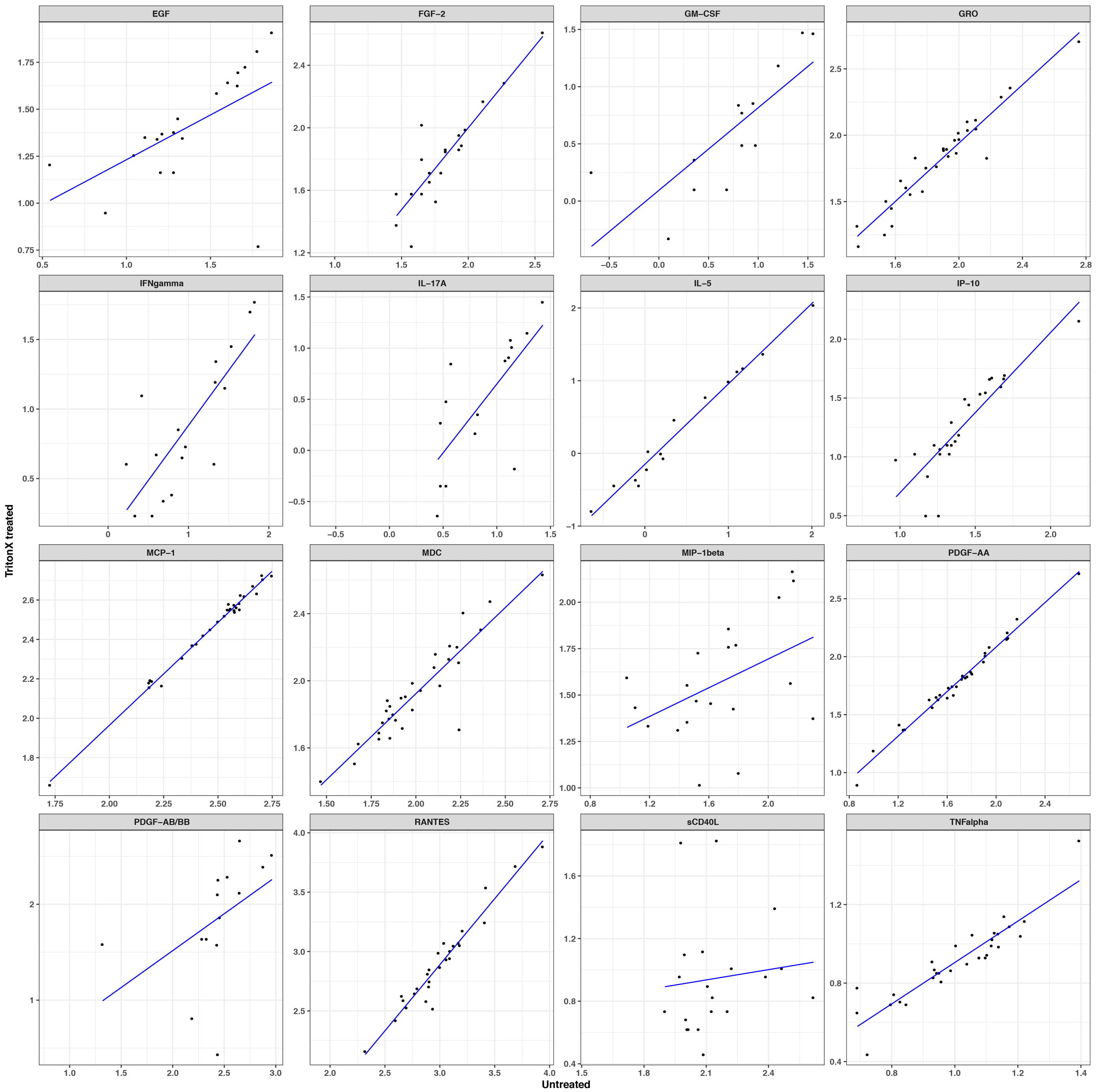

B

Analyte	n	t	P value	r	r2
MCP-1	31	55.3039	5.947E-31	0.9953	0.9906
PDGF-AA	31	42.6379	1.026E-27	0.9921	0.9843
IL-5	15	24.6689	2.652E-12	0.9895	0.9791
RANTES	27	19.2684	1.626E-16	0.9679	0.9369
GRO	29	17.0322	5.722E-16	0.9565	0.9149
MDC	31	12.1933	6.139E-13	0.9148	0.8368
TNFalpha	31	12.1135	7.206E-13	0.9138	0.8350
FGF-2	21	9.0321	2.642E-08	0.9006	0.8111
IP-10	24	8.4688	2.264E-08	0.8748	0.7653
IFNgamma	17	5.2232	1.031E-04	0.8033	0.6452
GM-CSF	13	4.2112	1.458E-03	0.7856	0.6172
IL-17A	15	3.7125	2.607E-03	0.7174	0.5146
EGF	19	2.8077	1.211E-02	0.5629	0.3168
PDGF-AB/BB	14	1.8287	9.239E-02	0.4668	0.2179
MIP-1beta	20	2.1319	4.705E-02	0.4490	0.2016
sCD40L	20	0.4825	6.353E-01	0.1130	0.0128

Correlation of analytes detected by Luminex with or without Triton-X treatment in healthy donors. The strength of the correlation between untreated and Triton-X treated samples were tested using a series of Pearson correlation tests. (A) Pearson correlation scatter plots showing the log10 analyte concentrations for untreated samples in the x axis and the same Triton-X treated sample in the y axis. Each point represents one biological sample with the best fit line (determined via the least squares method) in blue. (B) Table showing the details of the Pearson correlation tests. Each row represents the correlation results for one analyte with the number of biological samples (n), t statistics (t), nominal P value (P value), Pearson correlation coefficient (r) and Pearson correlation coefficient squared (r2) shown.

Supplementary Table 1. Demographics and clinical outcomes of COVID-19 patients.

Variable	Acute samples				Recovered samples				Paired samples in acute and recovered			
	**All patients (n = 54)**	**No pneumonia (n = 19)**	**Pneumonia without hypoxia (n = 11)**	**Pneumonia with hypoxia (n = 24)**	**All patients (n = 28)**	**No pneumonia (n = 7)**	**Pneumonia without hypoxia (n = 8)**	**Pneumonia with hypoxia (n = 13)**	**All patients (n = 11)**	**No pneumonia (n = 0)**	**Pneumonia without hypoxia (n = 2)**	**Pneumonia with hypoxia (n = 9)**
**Demographics**												
Median age, years	48.0 (38.5-61.3)	37.0 (33.0-47.0)	52.0 (47.0-62.0)	60.5 (45.5-69.0)	52.0 (41.5-60.5)	30.0 (26.0-54.0)	51.0 (43.3-59.8)	56.0 (50.5-64.5)	59.0 (50.0-66.0)	-	59.0 (56.0-62.0)	59.0 (42.0-67.5)
Sex, male (%)	50 (98.0)	18 (94.7)	11 (100.0)	21 (87.5)	19 (67.9)	4 (57.1)	6 (75.0)	9 (69.2)	10 (90.9)	-	2 (100.0)	8 (88.9)
Ethnicity (Chinese)	21 (38.9)	6 (31.6)	4 (36.4)	11 (45.8)	21 (75.0)	5 (71.4)	7 (87.5)	9 (69.2)	6 (54.6)	-	1 (50.0)	5 (55.6)
**Comorbidities**												
Diabetes	9 (16.7)	1 (5.3)	1 (9.1)	6 (25.0)	8 (28.6)	0 (0.0)	1 (12.5)	7 (53.9)	3 (27.3)	-	0 (0.0)	3 (33.3)
Hypertension	14 (25.9)	3 (15.8)	0 (0.0)	11 (45.8)	10 (35.7)	0 (0.0)	2 (25.0)	8 (61.5)	5 (45.5)	-	0 (0.0)	5 (55.6)
**Clinical outcome**												
Pneumonia with abnormal chest X ray (%)	35 (64.8)	0 (0.0)	11 (100.0)	24 (100.0)	21 (75.0)	0 (0.0)	8 (100.0)	13 (100.0)	11 (100.0)	-	2 (100.0)	9 (100.0)
Required supplemental oxygen (%)	24 (44.4)	0 (0.0)	0 (0.0)	24 (100.0)	13 (46.4)	0 (0.0)	0 (0.0)	13 (100.0)	9 (81.8)	-	0 (0.0)	9 (100.0)
ICU care (%)	15 (27.8)	0 (0.0)	0 (0.0)	15 (62.5)	9 (32.1)	0 (0.0)	0 (0.0)	9 (69.2)	5 (45.5)	-	0 (0.0)	5 (55.6)
Venous thromboembolism	3 (5.6)	0 (0.0)	0 (0.0)	3 (12.5)	-	-	-	-	0 (0.0)	-	0 (0.0)	0 (0.0)
Type 2 MI	3 (5.6)	0 (0.0)	0 (0.0)	3 (12.5)	-	-	-	-	0 (0.0)	-	0 (0.0)	0 (0.0)
*Bacterial co-infection (diagnosed after facs acquisition, no co-infection at the time of facs acquisition)	7 (13.0)	1 (5.3)	0 (0.0)	6 (25.0)	-	-	-	-	0 (0.0)	-	0 (0.0)	0 (0.0)
**Interval between symptom onset and sample acquisition (days pio)**	7.0 (2.0-11.0)	2.0 (2.0-5.5)	5.5 (2.3-8.8)	9.0 (6.3-12.0)	30.0 (22.5-35.8)	29.0 (18.0-39.0)	31.5 (29.3-34.0)	27.0 (21.5-38.5)	[a]12.0 (8.0-14.0)<br>[b]30.0 (22.0-35.0)	-	[a]9.0 (7.0-11.0)<br>[b]25.5 (21.0-30.0)	[a]12.0 (7.0-13.0)<br>[b]30.0 (24.0-38.0)

The values shown are based on available data. Categorical variables are shown as frequency (%). Continuous variables are shown median (IQR). *Types of bacterial co-infection include S.lugdunensis bloodstream infection, E.coli pneumonia with bloodstream infection, Enterobacter VAP, P.aeruginosa/K.pneumonia VAP, S.maltophilia VAP, methicillin-susceptible S. aureus VTE and left heel abscess. [a]Acute sample; [b]Recovered sample. MI, myocardial infarction; ICU, intensive care unit; IQR, Interquartile range; VAP, Ventilator-associated pneumonia; VTE, venous thromboembolism. [a]acute days pio, [b]recovered days pio

Supplementary Table 2. Demographics of healthy controls.

Variable	**Healthy controls (n = 19)**
**Demographics**	
Mean age, years	36 (10)
Sex, male (%)	10 (52.6)
Ethnicity (Chinese)	12 (63.2)
The values shown are based on available data. Categorical variables are shown as frequency (%). Continuous variables are shown mean (SD).	

Supplementary Table 3: Flow cytometry antibodies per panels

- **Panel A (100ul whole blood):**

No.	Marker	Colour	Volume (μL)	Clone	Cat. No.	Lot number	Vendor
1	CD45	BV786	2.5	HI30	304048	B284678	BioLegend
2	CD14	PE-CF594	1.5	MOP9	562335	9276099	BD Biosciences
3	CD16	APC Cy7	1.5	3G8	302018	B288665	BioLegend
4	CD19	SB600	2.5	SJ25C1	63-0198-42	2179717	eBioscience
5	CD11b	BV510	1.5	ICRF44	563098	9346006	BD Biosciences
6	CD33	PE-Cy7	1	WM-53	25-0338-42	E10580-1456	eBioscience
7	CD169	PE	1.5	7-239	346004	B272223	Biolegend
8	HLA-DR	AF700	1.5	L243	307626	B306020	Biolegend
9	CD3	APC	1.5	UCHT1	300439	B205424	Biolegend
10	CD56	FITC	5	MEM-188	304604	B291455	Biolegend
11	CD11c	BV650	2.5	B-ly6	563404	8187674	BD Biosciences
12	CD86	BV421	2	2331	562432	8337991	BD Horizon
13	CD123	BUV395	2	7G3	564195	9337379	BD Horizon
14	CD66b	PerCP cy5.5	2	G10F5	305108	B204076	Biolegend

- **Panel B (100ul whole blood):**

No.	Marker	Colour	Volume (μL)	Clone	Cat No.	Lot number	Vendor
1	CD3	FITC	1	UCHT1	11-0038-42	2007254	eBioscience
2	CD4	BV650	2	SK3	563875	9107661	BD Horizon
3	CD8	V500	2	RPA-T8	560774	4052849	BD Biosciences
4	CD45RA	PerCP-Cy5.5	2	HI100	304122	B284187	Biolegend
5	CD27	APC	2	O323	17-0279-42	2168714	eBioscience
6	CD25	PE-Cy7	2	M-A251	557741	9301660	BD Biosciences
7	CD127	BUV737	2	HL-7R-M21	564300	9289985	BD Biosciences
8	CD38	BUV395	2	HB7	563811	9155743	BD Biosciences
9	CD56	PE	2	AF12-7H3	130-098-755	5160830148	Miltenyi Biotec
10	CD16	AF700	2	3G8	302036	B266048	Biolegend
11	Vδ1 TCR	APC-Vio770	1	REA173	130-120-438	5200304105	Miltenyi Biotec
12	Vδ2 TCR	BV711	2	B6	331412	B285901	Biolegend
13	VA7.2 TCR	BV605	2	3C10	351720	B275819	Biolegend

14	CD161	BV786	2	HP-3G10	339930	B258781	Biolegend
15	CD19	PE-CF594	2	HIB19	562321	B277541	BD Biosciences
16	CD57	PB	0.5	HCD57	322316	B270598	Biolegend

- **Panel C (100ul whole blood):**

No.	Marker	Colour	Volume (μL)	Clone	Cat. No.	Lot number	Vendor
1	CD45RA	PerCP Cy5.5	1	HI100	304122	B284187	Biolegend
2	CD10	FITC	1	HI10a	312208	B270343	Biolegend
3	CD11b	PE-Cy7	1	ICRF44	25-0118-42	1983204	eBioscience
4	CD49d	PE-CF594	1	9F10	563645	9261644	BD Biosciences
5	Siglec8	PE	1	7C9	347104	B274554	Biolegend
6	CD8	BV786	0.5	RPA-T8	563823	9344069	BD Biosciences
7	CD4	BV650	1	RPA-T4	300536	B292888	Biolegend
8	CD16	BV605	1	3G8	563172	9179026	BD Horizon
9	CD3	V500	5	UCHT1	561416	9191445	BD Biosciences
10	CD66b	BV421	1	G10F5	562940	9308264	BD Biosciences
11	HLA-DR	APC-H7	0.5	G46-6	561358	9078946	BD Biosciences
12	CCR3	AF647	2	5E8	310710	B220159	Biolegend
13	CD38	BUV395	3	HB7	563811	9155743	BD Biosciences
14	CD27	BUV737	2	L128	564301	9109918	BD Biosciences
