# Supplementary material for: Whole blood immunophenotyping uncovers immature neutrophil-to-VD2 T-cell ratio as an early marker for severe COVID-19
Source: Nat Commun. 2020 Oct 16;11:5243. doi: 10.1038/s41467-020-19080-6 (PMC7568554; doi:10.1038/s41467-020-19080-6)
Supplement: Supplementary file 3 — Description of Additional Supplementary Files [file 41467_2020_19080_MOESM3_ESM.pdf]

Description of Additional Supplementary Files

File Name: Supplementary Data 1
Description: Excel file containing detailed information of the statistical tests performed, multiple correction and exact or adjusted p value when available for figures 1, 2, 3 and 5a.

File Name: Supplementary Software 1
Description: zip file containing the R scripts used to generate the heatmaps and the UMAPs of figure 1 and 2; as well as the R script used to generate the correlation matrix in Figure 4.
