# Supplementary material for: Whole blood immunophenotyping uncovers immature neutrophil-to-VD2 T-cell ratio as an early marker for severe COVID-19
Source: Nat Commun. 2020 Oct 16;11:5243. doi: 10.1038/s41467-020-19080-6 (PMC7568554; doi:10.1038/s41467-020-19080-6)
Supplement: Supplementary file 6 — Reporting Summary [file 41467_2020_19080_MOESM6_ESM.pdf]

nature research

Corresponding author(s): Lisa FP Ng

Last updated by author(s): Sep 6, 2020

Reporting Summary

Nature Research wishes to improve the reproducibility of the work that we publish. This form provides structure for consistency and transparency in reporting. For further information on Nature Research policies, see our Editorial Policies and the Editorial Policy Checklist.

Statistics

For all statistical analyses, confirm that the following items are present in the figure legend, table legend, main text, or Methods section.

n/a	Confirmed	
☐	☒	The exact sample size (*n*) for each experimental group/condition, given as a discrete number and unit of measurement
☐	☒	A statement on whether measurements were taken from distinct samples or whether the same sample was measured repeatedly
☐	☒	The statistical test(s) used AND whether they are one- or two-sided<br>*Only common tests should be described solely by name; describe more complex techniques in the Methods section.*
☐	☒	A description of all covariates tested
☐	☒	A description of any assumptions or corrections, such as tests of normality and adjustment for multiple comparisons
☐	☒	A full description of the statistical parameters including central tendency (e.g. means) or other basic estimates (e.g. regression coefficient) AND variation (e.g. standard deviation) or associated estimates of uncertainty (e.g. confidence intervals)
☐	☒	For null hypothesis testing, the test statistic (e.g. F, t, r) with confidence intervals, effect sizes, degrees of freedom and P value noted<br>*Give P values as exact values whenever suitable.*
☒	☐	For Bayesian analysis, information on the choice of priors and Markov chain Monte Carlo settings
☒	☐	For hierarchical and complex designs, identification of the appropriate level for tests and full reporting of outcomes
☐	☒	Estimates of effect sizes (e.g. Cohen's d, Pearson's r), indicating how they were calculated

Our web collection on statistics for biologists contains articles on many of the points above.

Software and code

Policy information about availability of computer code

Data collection	Flow cytometry data was collected using BD LSRII 5 laser configuration using automatic compensations running BD FACS Diva Software version 8.0.1 (build 2014 07 03 11 47), Firmware version 1.14 (BDLSR II), CST version 3.0.1, PLA version 2.0.<br>Patient clinical information was collected using Excel for Mac version 16.16.8 (Microsoft, USA).<br>Luminex data acquisition was performed on FLEXMAP® 3D (Luminex) using xPONENT® 4.0 (Luminex) software. Data analysis was done on Bio-Plex ManagerTM 6.1.1 (Bio-Rad).
Data analysis	Data was analyzed using Flowjo version 10.6.1, GraphPad Prism versions 7.03 to 8.3.0 (GraphPad Software, San Diego, CA, USA), Excel for Mac version 16.16.8 (Microsoft, USA), RStudio v3.5.2 using cytofkit2 R Packages and custom R scripts available in supplementary file 2.

For manuscripts utilizing custom algorithms or software that are central to the research but not yet described in published literature, software must be made available to editors and reviewers. We strongly encourage code deposition in a community repository (e.g. GitHub). See the Nature Research guidelines for submitting code & software for further information.

Data

Policy information about availability of data

All manuscripts must include a data availability statement. This statement should provide the following information, where applicable:

- Accession codes, unique identifiers, or web links for publicly available datasets
- A list of figures that have associated raw data
- A description of any restrictions on data availability

Source data are provided with this paper. Custom R scripts are available in Supplementary File 2. Other data not made publicly available due to protection of patients' confidentiality can be obtained upon request to the corresponding author.

Field-specific reporting

Please select the one below that is the best fit for your research. If you are not sure, read the appropriate sections before making your selection.

[x] Life sciences [ ] Behavioural & social sciences [ ] Ecological, evolutionary & environmental sciences

For a reference copy of the document with all sections, see nature.com/documents/nr-reporting-summary-flat.pdf

Life sciences study design

All studies must disclose on these points even when the disclosure is negative.

Sample size	No sample size calculation was performed prior to the study. Samples were acquired upon successful recruitment of patients and healthy donors.<br>Acute SARS-CoV-2: 54 unique patients<br>Recovered from SARS-CoV-2: 28 unique patients<br>Healthy donors: 19 unique healthy donors
Data exclusions	For some samples, data from UV channels of the flow cytometer (cell counts and MFI) were excluded from the analysis on the basis that the UV laser failed during that acquisition day.
Replication	Whole blood was acquired at one time without technical replicate for each patients. Patients did not provide duplicate samples during collection. Acute sample storage was not permitted by the Singapore Health and Safety Board also preventing replication on stored material. Flow cytometer was calibrated before and after each acquisition using Rainbow Calibration Particles, 8 peaks (Biolegend ref 422903) to validate consistent acquisitions during the entire course of the study.
Randomization	Acquisition was performed on the basis of recruitment after informed consent. Randomization was not performed as all successfully recruited patients were acquired.
Blinding	Investigators were blinded to clinical parameters during acquisition and analysis of flow cytometry data. Clinical parameters were made available after in order to allocate groups and perform secondary analysis.

Reporting for specific materials, systems and methods

We require information from authors about some types of materials, experimental systems and methods used in many studies. Here, indicate whether each material, system or method listed is relevant to your study. If you are not sure if a list item applies to your research, read the appropriate section before selecting a response.

Materials & experimental systems

n/a	Involved in the study	
[ ]	[x]	Antibodies
[x]	[ ]	Eukaryotic cell lines
[x]	[ ]	Palaeontology and archaeology
[x]	[ ]	Animals and other organisms
[ ]	[x]	Human research participants
[x]	[ ]	Clinical data
[x]	[ ]	Dual use research of concern

Methods

n/a	Involved in the study	
[x]	[ ]	ChIP-seq
[ ]	[x]	Flow cytometry
[x]	[ ]	MRI-based neuroimaging

Antibodies

Antibodies used

• Panel A (100ul whole blood):
Marker Clone Cat. No. Lot number Vendor
CD45 HI30 304048 B284678 BioLegend
CD14 MOP9 562335 9276099 BD Biosciences
CD16 3G8 302018 B288665 BioLegend
CD19 SJ25C1 63-0198-42 2179717 eBioscience
CD11b ICRF44 563098 9346006 BD Biosciences
CD33 WM-53 25-0338-42 E10580-1456 eBioscience
CD169 7-239 346004 B272223 Biolegend
HLA-DR L243 307626 B306020 Biolegend
CD3 UCHT1 300439 B205424 Biolegend
CD56 MEM-188 304604 B291455 Biolegend
CD11c B-ly6 563404 8187674 BD Biosciences
CD86 2331 562432 8337991 BD Horizon

CD123 7G3 564195 9337379 BD Horizon
CD66b G10F5 305108 B204076 Biolegend

• Panel B (100ul whole blood):
Marker Clone Cat No. Lot number Vendor
CD3 UCHT1 11-0038-42 2007254 eBioscience
CD4 SK3 563875 9107661 BD Horizon
CD8 RPA-T8 560774 4052849 BD Biosciences
CD45RA HI100 304122 B284187 Biolegend
CD27 O323 17-0279-42 2168714 eBioscience
CD25 M-A251 557741 9301660 BD Biosciences
CD127 HL-7R-M21 564300 9289985 BD Biosciences
CD38 HB7 563811 9155743 BD Biosciences
CD56 AF12-7H3 130-098-755 5160830148 Miltenyi Biotec
CD16 3G8 302036 B266048 Biolegend
V1 TCR REA173 130-120-438 5200304105 Miltenyi Biotec
V2 TCR B6 331412 B285901 Biolegend
VA7.2 TCR 3C10 351720 B275819 Biolegend
CD161 HP-3G10 339930 B258781 Biolegend
CD19 HIB19 562321 B277541 BD Biosciences
CD57 HCD57 322316 B270598 Biolegend

• Panel C (100ul whole blood):
Marker Clone Cat. No. Lot number Vendor
CD45RA HI100 304122 B284187 Biolegend
CD10 HI10a 312208 B270343 Biolegend
CD11b ICRF44 25-0118-42 1983204 eBioscience
CD49d 9F10 563645 9261644 BD Biosciences
Siglec8 7C9 347104 B274554 Biolegend
CD8 RPA-T8 563823 9344069 BD Biosciences
CD4 RPA-T4 300536 B292888 Biolegend
CD16 3G8 563172 9179026 BD Horizon
CD3 UCHT1 561416 9191445 BD Biosciences
CD66b G10F5 562940 9308264 BD Biosciences
HLA-DR G46-6 561358 9078946 BD Biosciences
CCR3 5E8 310710 B220159 Biolegend
CD38 HB7 563811 9155743 BD Biosciences
CD27 L128 564301 9109918 BD Biosciences

Validation

Antibodies were purchased as validated from each manufacturer for their human targets.
Each lot received from the manufacturer was validated in-house on healthy donor blood samples by flow cytometry to validate the dilution used in this study.

Human research participants

Policy information about studies involving human research participants

Population characteristics

healthy donors (n=19): Mean age, years (SD) 36 (10) ; Sex, male (%) 10 (52.6) ; Ethnicity (% Chinese) 12 (63.2)
Acute patients (n=54): Median age, years (range) 48.0(38.5-61.3) ; Sex, male (%) 50 (98.0) ; Ethnicity (% Chinese) 21 (38.9)
Recovered patients (n=28): Median age, years (range) 52.0 (41.5-60.5) ; Sex, male (%) 19 (67.9) ; Ethnicity (% Chinese) 21 (75.0)

Recruitment

Recruitment of SARS-CoV-2 PCR positive patient was performed with informed consent at National Centre for Infectious Disease in SIngapore. No selection was performed, all consenting patients were recruited.
Healthy donors were recruited internally at the Singapore Immunology Network. Selection was based on availability and consent.

Ethics oversight

Written informed consent was obtained from participants in accordance with the tenets of the Declaration of Helsinki. For COVID-19 blood/plasma collection, “A Multi-centred Prospective Study to Detect Novel Pathogens and Characterize Emerging Infections (The PROTECT study group)”, a domain specific review board (DSRB) evaluated the study design and protocol, which was approved under study number 2012/00917 the National Healthcare Group (NHG). Healthy volunteers samples were obtained under the following IRB “Study of blood cell subsets and their products in models of infection, inflammation and immune regulation” under the number 2017/2806 by the SingHealth Centralised Institutional Review Board (CIRB).

Note that full information on the approval of the study protocol must also be provided in the manuscript.

Flow Cytometry

Plots

Confirm that:

☒ The axis labels state the marker and fluorochrome used (e.g. CD4-FITC).

☒ The axis scales are clearly visible. Include numbers along axes only for bottom left plot of group (a 'group' is an analysis of identical markers).

☒ All plots are contour plots with outliers or pseudocolor plots.

☒ A numerical value for number of cells or percentage (with statistics) is provided.

Methodology

Sample preparation	Whole blood from CPT or ETDA tube was stained directly
Instrument	BD LSRII 5 laser configuration using automatic compensations
Software	BD FACS Diva Software version 8.0.1 (build 2014 07 03 11 47), Firmware version 1.14 (BDLSR II), CST version 3.0.1, PLA version 2.0
Cell population abundance	cell counts were calculated using counting beads as indicated in the methods
Gating strategy	Panel A:<br>SSC-A vs CD45 allows identification of CD45+ population. From that, FSC-A vs FSC-H single cells are obtained, a second gating for ssc-a vs cd45 allow to clean the cd45+ population. Next high SSC-A cd66b+ population is the granulocytes from which can be gated the neutrophils as cd16+. From the low SSC-A population, CD19 vs CD3 allows to separate the B-cells (CD19+) and the T-cells (CD3+) and the double negative (DN) population. The DN population can then be used to gate the NK cells as SSC-A low, CD56+ population, those can the be separated into the CD56bright CD16- ; CD56int Cd16- and CD56int CD16+ populations. The non-NK cells can then be use to gate the CD33-CD123+ pDCs (which can then be cleaned into true pDCs as SSC-A low HLA-DR+) and the Basophils (CD33int, CD123+). The non-pDCs non-Basophils population can then be used to gate the Classical(CD14+CD16-)/intermediate(CD14+CD16+) and non-classical monocytes(CD14int CD16+) using CD14 vs CD16. The DC population (CD11c+HLA-DR+) can then be separated from the CD14-CD16- cells using CD11c vs HLA-DR. From all those population the activation markers can be investigated for % positive and gMFI.<br><br>Panel B:<br>SSC-A low vs FSC-A allows to gate the lymphocyte and monocyte populations. From that, FSC-A vs FSC-H single cells are obtained. SSC-A low CD3+ T-cell population can then be gated. gamma delta T-cells can then be separated as VD1+ or VD2+. VD1-VD2- T-cells can be used to gate the MAIT cells (CD161+ VA7.2+) and the non-MAIT cells, which are then separated into CD4+ or CD8+ or DN T-cells. The CD4 T-cells can then be separate into Tregs (CD25+, CD127-) and effector CD4 T-cells (rest of the CD4+). From the CD3- population, we can gate the B-cells (CD19+) and the NK cells (CD56+). Plasmablast can be separated from the B-cell population using the CD38high Cd27high gate, while the NK population can ge divided in the 3 populations (as in panel A). Naive, memory and effector T-cell compartments can be investigated using the standard CD45RA vs CD27 quadrant methods. From all those population the activation and phenotypic markers can be investigated for % positive and gMFI.<br><br>Panel C:<br>Single cells are first gated using the SSC-A vs SSC-H gate, and then cells are gated using the FSC-A and SSC-A gate. From that, the T-cells containing population (CD3+) and a non-T-cell containing population (CD3-) can be separated. In the CD3+ population, CD4+ and CD8+ T-cell population can be gated and their HLA-DR expression assessed. From the CD3- population, the CD66B vs CCR3 allows to gate the CCR3+CD66B- population, the CCR3+ CD66B+ eosinophil population (which is then cleaned in true eosinophils as the CD16- population), and the CCR3- population (non-eosinophil). These CCR3- cells and then gated as neutrophils in the CD66B+ CD11b+ gate. Next, pre-neutrophils can be gated as CD49dintCD16- and non pre-neutrophils can be gated as CD16+ CD49d-. These can then be separated using CD16 vs CD10 as mature neutrophils (CD10+) and immature neutrophils (CD10-). In this gate CD16 expression can be variable depending on the sample type (infected vs healthy).

☒ Tick this box to confirm that a figure exemplifying the gating strategy is provided in the Supplementary Information.
